# Pulmonary nocardiosis following *COVID-19* in a patient with idiopathic pulmonary fibrosis and lung transplantation: a case report

**DOI:** 10.3389/fmed.2023.1266857

**Published:** 2023-09-12

**Authors:** Liming Cao, Yilan Sun, Fei Chen

**Affiliations:** Cancer Center, Department of Pulmonary and Critical Care Medicine, Zhejiang Provincial People’s Hospital (Affiliated People’s Hospital), Hangzhou Medical College, Hangzhou, Zhejiang, China

**Keywords:** pulmonary nocardiosis, *COVID-19*, idiopathic pulmonary fibrosis, lung transplantation, immunosuppression

## Abstract

**Background:**

Nocardiosis is an opportunistic infection that primarily affects immunocompromised patients. Pulmonary nocardiosis is the most prevalent form, but can also spread to other organs. Potential causes contributing to opportunistic infection may include immunosuppression and disruption of tight junctions, both of which can result from *COVID-19*.

**Case presentation:**

We reported a case of a 68-year-old male patient who presented with a 10-day history of fever, cough, and productive sputum. Upon physical examination, velcro rales were detected in the right lung, while breath sounds in the left lung were clear. The patient had previously undergone left lung transplantation due to idiopathic pulmonary fibrosis four years ago. He was initially hospitalized and treated for *COVID-19* but was readmitted due to worsening symptoms. Subsequently, pulmonary nocardiosis was diagnosed utilizing metagenomic next-generation sequencing of bronchoalveolar lavage fluid. The above-mentioned condition was improved following treatment with cancidas and linezolid. Now, he is under regular follow-up.

**Conclusion:**

This case highlights the complexity of *COVID-19* and the occurrence of secondary opportunistic infections, which require further investigation.

## Introduction

The incidence of Nocardia infections has gradually increased due to the growing number of immunocompromised patients, including those with tumors, organ transplantation, and on chronic steroid therapy (1, 2). This paper presents a pneumonia case of *Nocardia farcinica* infection involving multiple risk factors, including lung transplantation, post-*COVID-19* status, and steroid use.

### Case presentation

**Figure 1:**
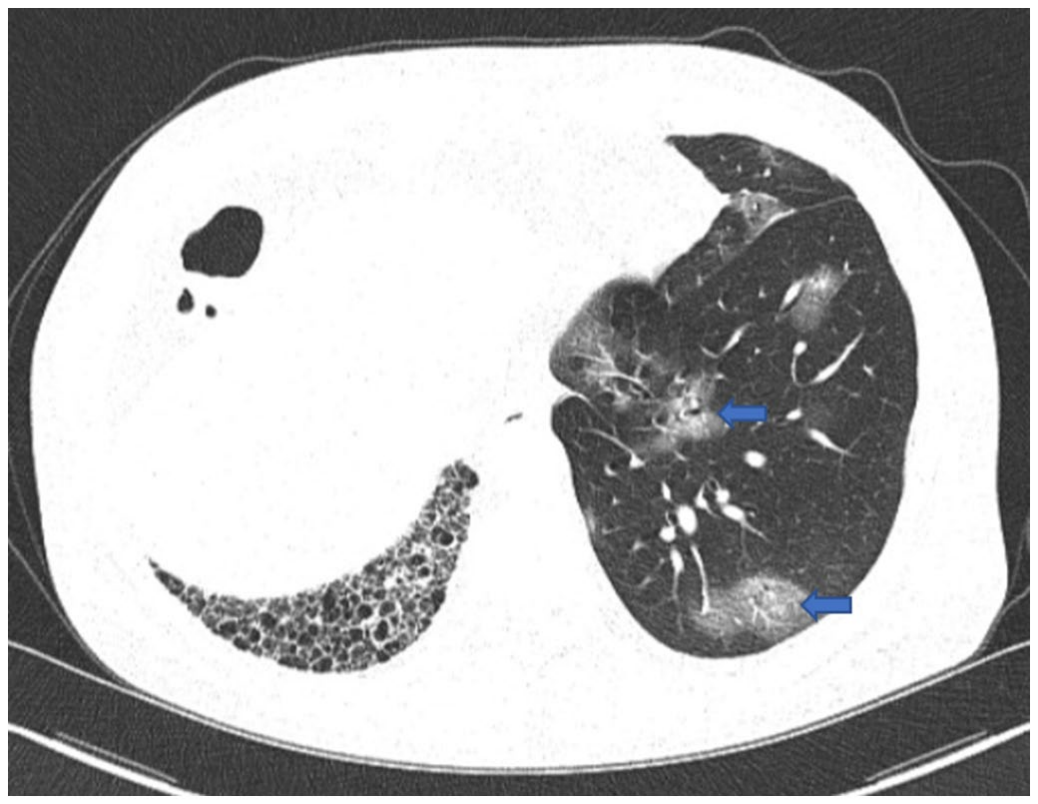
First chest CT image. The image showed *COVID-19* pneumonia with scattered ground-glass opacities in the left lung (blue arrow), and fibrosis and honeycomb changes in the right lung.

On December 29th, 2022, a 68-year-old male was admitted to the hospital with a complaint of fever, cough, and productive sputum persisting for 10 days. The patient developed a fever 10 days ago, with a maximum body temperature of 39.5°C. Additionally, the patient experienced coughing and sputum production but without hemoptysis, dyspnea, chest pain, or chest tightness. Despite receiving antipyretic treatments, the patient experienced recurring fever. Consequently, he sought medical attention at the pulmonary specialist clinic and was hospitalized. Four years ago, he received left lung transplantation due to idiopathic pulmonary fibrosis (IPF) and has since been prescribed tacrolimus, mycophenolate mofetil, and prednisone. During the physical examination, velcro rales were detected in the right lung, while the breath sounds in the left lung were found to be clear. In our department, the patient tested positive for *COVID-19* nucleic acid, with a 73.4 mg/L increase in C-reactive protein (CRP), while white blood cell (WBC) and neutrophil counts were within the normal range. Meanwhile, the analysis of lymphocyte subsets revealed a decrease in CD8^+^T cells and B cells, while CD4^+^T cells remained within the normal range. Other laboratory values were displayed in Table 1. Scattered ground-glass opacities were observed in the left lung on first chest CT imaging, along with fibrosis and honeycomb changes in the right lung (Figure 1). Based on the epidemiology of *COVID-19* in China, along with the patient’s medical history and positive examinations, a diagnosis of *COVID-19* pneumonia was made. The treatment involved the administration of Paxlovid and methylprednisolone intravenously and subsequently orally for 21 days, with a total dosage of approximately 400 mg. Simultaneously, tacrolimus was discontinued while the dosage of mycophenolate mofetil was reduced. Throughout his hospitalization, he did not require supplemental oxygen or mechanical ventilation. After the symptoms improved, he was discharged.

**Table 1:** Laboratory values on the first day of two hospitalizations.

Parameter	First hospitalization	Second hospitalization	Reference value
WBC(10^9^/L)	4.49	12.63	3.5–9.5
Neutrophil(10^9^/L)	2.85	9.95	1.8–6.3
lymphocyte (10^9^/L)	1.39	1.96	1.1–3.2
CD3 + CD4 + (10^6^/L)	599	1,040	537–1,282
CD3 + CD8 + (10^6^/L)	171	221	258–1,042
CD19(10^6^/L)	96	205	173–447
CRP(mg/L)	73.4	133.8	≤10
Procalcitonin (ng/ml)	0.07	0.14	≤0.25
ESR(mm/h)	26	51	<43

WBC, white blood cell count; CRP, C-reactive protein; ESR, erythrocyte sedimentation rate.

**Figure 2:**
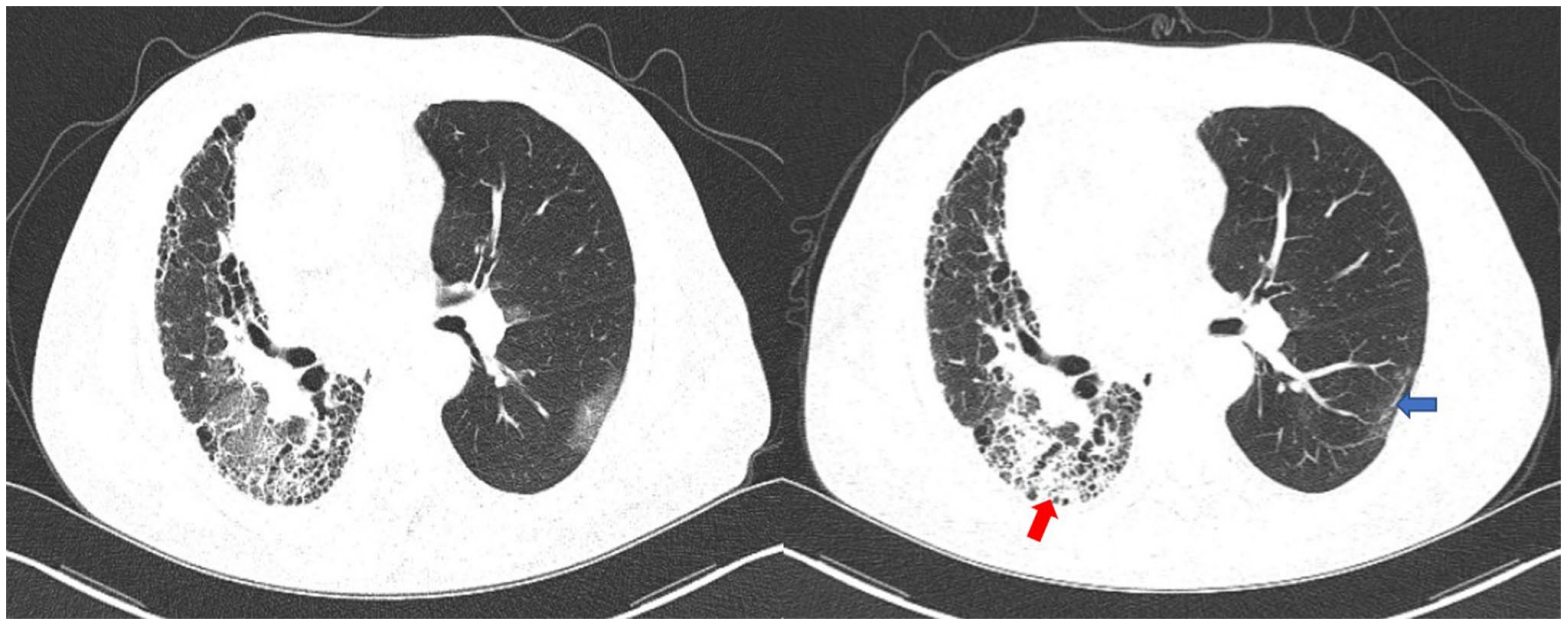
The first chest CT scan (left) and the second chest CT scan (right). The second chest CT image showed consolidation superimposed to pre-existing fibrotic changes on the lower lobe of the right lung (red arrow). The *COVID-19*-related ground-glass opacities on the left lung disappeared on the second CT, with residual tiny scars (blue arrow).

Two weeks later, the patient was readmitted to the hospital due to an exacerbation of cough and expectoration. The patient tested positive for *COVID-19* nucleic acid once more, with a significant increase in WBC, neutrophils, and CRP levels (Table 1). The second pulmonary CT scan showed consolidation superimposed to pre-existing fibrotic changes on the lower lobe of the right lung, which was absent in the first chest CT scan. Additionally, the *COVID-19*-related ground-glass opacities on the left lung disappeared on the second CT, with residual tiny scars (Figure 2). Given the possibility of bacterial or fungal co-infection, we used piperacillin sodium-tazobactam sodium intravenously (4.5g q8h) for a week and caspofungin (70 mg loading dose on day 1, followed by 50 mg daily) for 2 weeks.

**Figure 3:**
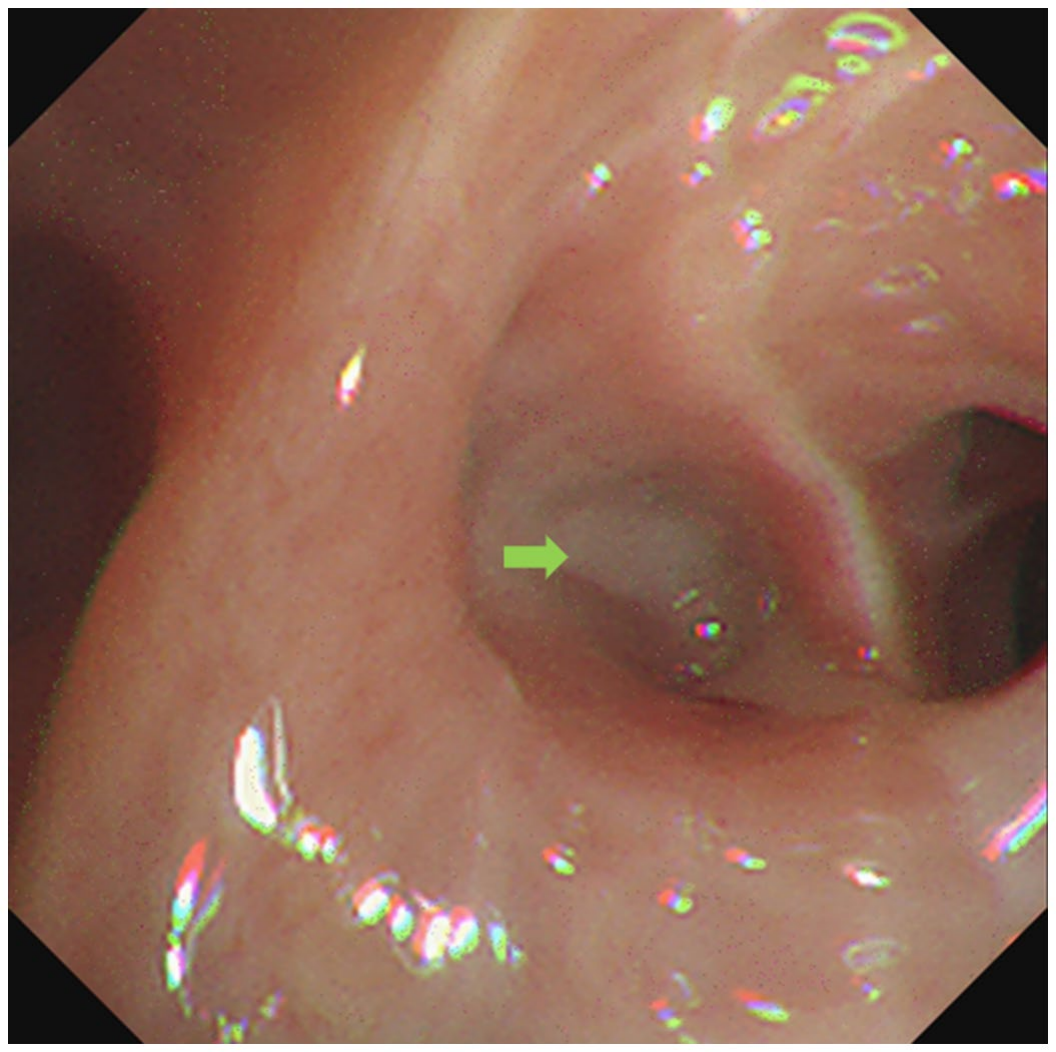
Bronchoscope showed respiratory secretion in the dorsal branch of the right lower lung (green arrow).

On the second day of hospitalization, we conducted a bronchoscopy. During bronchoscopy, narrowing of the lumen in the dorsal branch of the right lower lung with visible secretions was observed; no abnormalities were detected in other lobes (Figure 3). Metagenomic next-generation sequencing (mNGS) of alveolar lavage fluid identified the presence of *Nocardia farcinica*, an opportunistic pathogen commonly associated with infections in immunocompromised patients. Following a definitive diagnosis, linezolid (0.6 g q12h) was given intravenously for 2 weeks, while molnupiravir (0.8 g bid) for five days was prescribed specifically for the *COVID-19* coronavirus. Both tacrolimus and mycophenolate mofetil were administered as per standard protocol. Finally, the inflammatory index progressively decreased, leading to an improvement in the condition. After discharge, the patient switched to oral treatment of trimethoprim-sulfamethoxazole.

**Figure 4:**
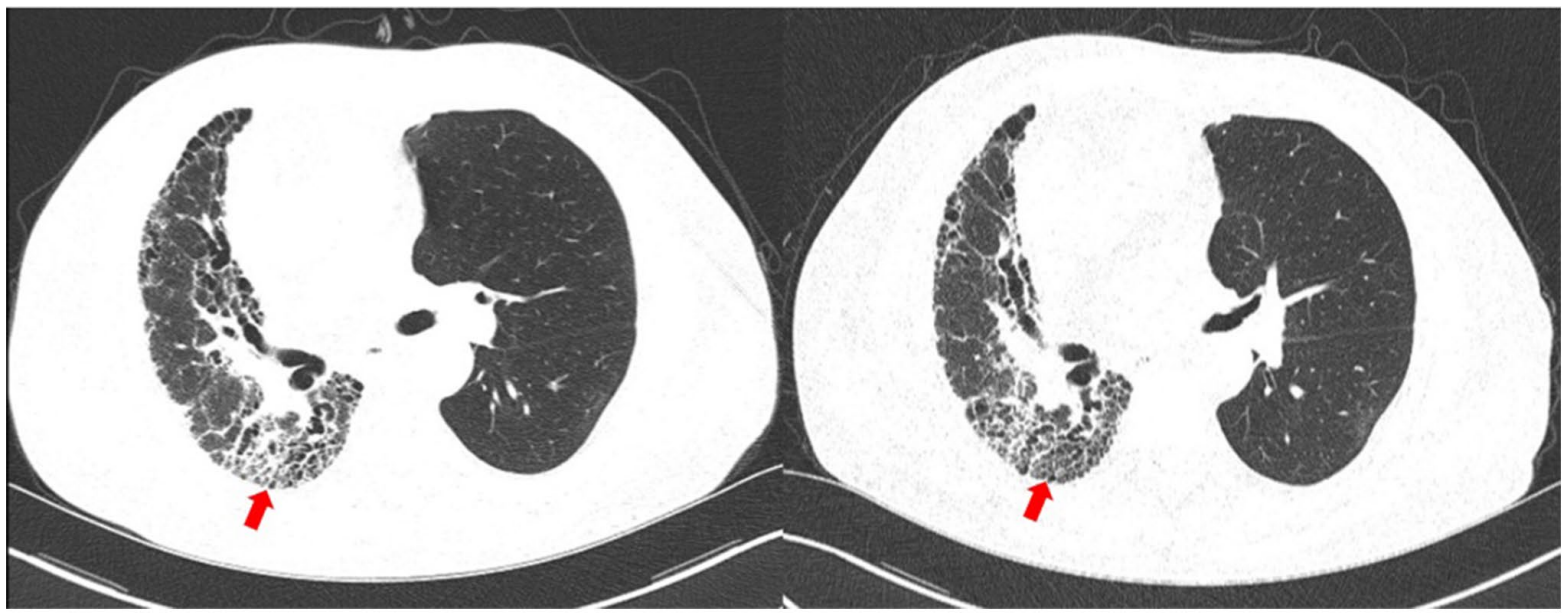
Chest CT upon 14 days (left) and 85 days (right) after the diagnosis. The nocardiosis lesions in the right lung were gradually absorbed (red arrow).

**Figure 5:**
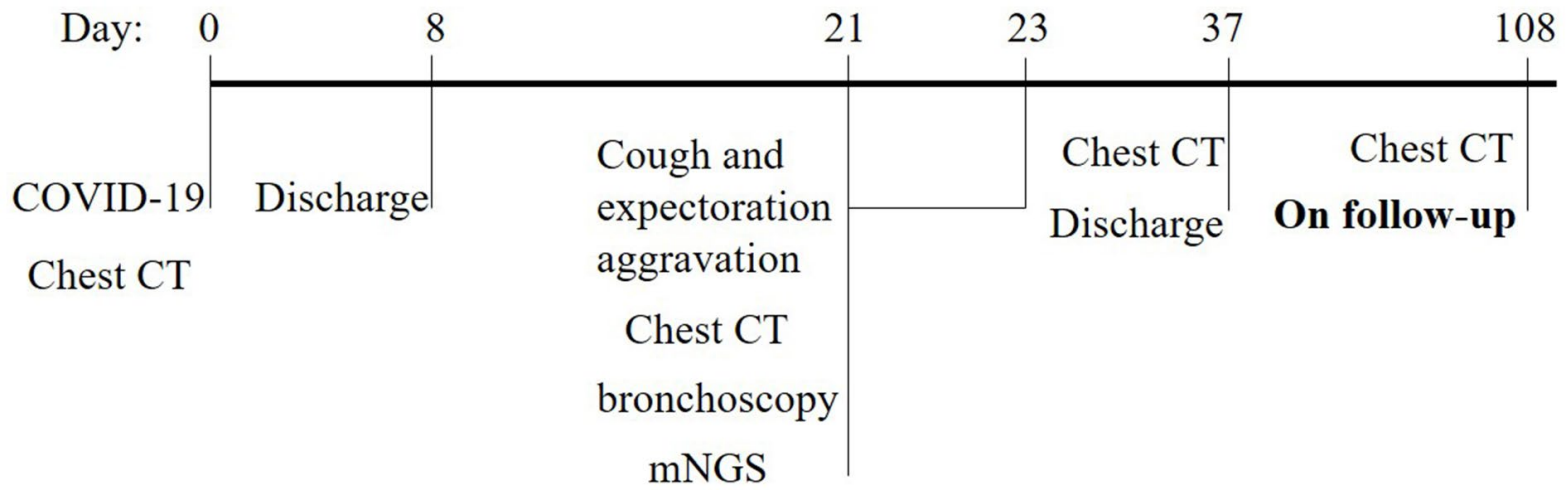
Timeline of medical history.

Upon 14 days and 85 days after the diagnosis, the patient underwent chest CT examination, which displayed nocardiosis lesions in the right lung were gradually resolved (Figure 4). The patient is currently undergoing regular follow-up at the clinic. The medical timeline is listed in Figure 5.

## Discussion

Nocardia is a Gram-positive bacterium that is ubiquitous in nature, primarily found in soil and humid environments. It frequently causes infections in immunocompromised patients, including those undergoing solid organ transplantation, suffering from chronic lung disease, diabetes, malignancy, or using long-term steroid therapy (3–6). Pulmonary nocardiosis is the most common type, presenting with nonspecific symptoms such as fever, cough, and chest pain, as well as CT imaging findings of lung consolidation, nodules/masses, ground-glass opacity, and centrilobular nodules. Delayed diagnosis contributes to its high mortality rate (5, 7, 8).

This patient had numerous risk factors, including immunosuppression caused by daily antirejection therapy due to lung transplantation, *COVID-19* infection, and corticosteroid therapy. Previous studies have identified Nocardia as a prevalent pathogen in chronic pulmonary infections among patients with IPF, yet it has not been widely recognized as a contributing risk factor (9, 10). Lung transplant recipients, classified as a specific subgroup of immunocompromised patients, are particularly susceptible to Nocardia as a significant pathogen that primarily affects the native lung in cases of unilateral lung transplantation (11). Previous study on pulmonary nocardiosis following lung transplantation has largely reported that a majority cases were attributed to *N. farcinica, N. nova*, and *N. asteroids* (6).

Given the patient’s stable condition following lung transplantation, we hypothesized that the disease may be attributed to *COVID-19* infection and corticosteroid therapy used for its treatment. Corticosteroids can result in secondary infections, such as fungal, viral, mycobacterial, and Nocardia (12, 13). A retrospective study has conclusively established a correlation between glucocorticoid therapy and Nocardia infection (14).

However, *COVID-19* can also induce an immunocompromised state, as observed in a case of encephalic nocardiosis occurring after *COVID-19*, even in the absence of steroid use. Nocardiosis has been reported in cases where the primary immune response is predominantly mediated by CD8^+^T cells, while B lymphocytes and humoral immunity may play a lesser role (15). While in *COVID-19* patients, lymphopenia accompanied by a severe decline in CD4^+^ and CD8^+^T cells, B cells, and innate immune cells is a common feature, and this patient specifically exhibited decreased CD8^+^T cells (16). A subsequent study has shown that lymphopenia may be associated with reduced levels of protein tyrosine phosphatase receptor type C, leptin, and tartrate-resistant acid phosphatase type 5 (17). However, the duration of this immune disorder after *COVID-19* is unclear (18). Furthermore, impairment of the tight junction complex can occur, creating conditions for bacterial attachment (17). These changes may impact the response of *COVID-19* patients to opportunistic bacterial infection caused by Nocardia.

## Conclusion

This case highlights the *COVID-19*-induced immunologic derangement, along with the role of glucocorticoids, which requires further investigation to elucidate the specific immune status. It is also vital to remain mindful of the potential for Nocardia opportunistic infection following *COVID-19*.

## Data availability statement

The original contributions presented in the study are included in the article/supplementary material, further inquiries can be directed to the corresponding author.

## Ethics statement

The studies involving humans were approved by Zhejiang Provincial People’s Hospital. The studies were conducted in accordance with the local legislation and institutional requirements. The participants provided their written informed consent to participate in this study.

Written informed consent was obtained from the individual(s) for the publication of any potentially identifiable images or data included in this article.

## Author contributions

LC: writing – original draft, writing – review and editing. YS: writing – review and editing. FC: writing – review and editing, writing – original draft.

## Funding

This work was financially supported by the Zhejiang Medical and Health Science and Technology Project (2019316221).

## Conflict of interest

The authors declare that the research was conducted in the absence of any commercial or financial relationships that could be construed as a potential conflict of interest.

## Publisher’s note

All claims expressed in this article are solely those of the authors and do not necessarily represent those of their affiliated organizations, or those of the publisher, the editors and the reviewers. Any product that may be evaluated in this article, or claim that may be made by its manufacturer, is not guaranteed or endorsed by the publisher.

## Abbreviations

IPF: idiopathic pulmonary fibrosis
mNGS: metagenomic next-generation sequencing
WBC: white blood cell
CRP: C-reactive protein
ESR: erythrocyte sedimentation rate
